# Long-term safety evaluation of Daxocox^®^ tablets (enflicoxib) in dogs after weekly oral administrations for seven months

**DOI:** 10.1186/s12917-021-02910-0

**Published:** 2021-06-03

**Authors:** Josep Homedes, Marta Salichs, Antonio Guzman

**Affiliations:** 1 Ecuphar Veterinaria SLU (Animalcare Group) Avgda, Río de Janeiro 60 – 66 planta 13, 08016 Barcelona, Spain; 2 Welab, Barcelona Science Park (PCB), Baldiri Reixac 4-8, Edifici Clúster II, 08028 Barcelona, Spain

**Keywords:** Enflicoxib, Daxocox, Safety, Dog, TAS

## Abstract

**Background:**

Daxocox® [Ecuphar/Animalcare Group] contains the selective COX-2 inhibitor enflicoxib, approved in the EU for the treatment of pain and inflammation associated with osteoarthritis in dogs. The safety of Daxocox^®^ was evaluated in a target animal safety study: Groups of 4 dogs per sex each were treated once weekly with placebo or Daxocox tablets at 1-, 3- and 5-times (1X, 3X and 5X) the maximum recommended therapeutic dose of enflicoxib (0, 4, 12 or 20 mg/kg, respectively). After an initial loading dose, dogs in the placebo control, 1X and 3X groups were administered for 32 weeks, and those in the 5X group were administered for 13 weeks. Dogs were subjected to daily food consumption measurements and clinical and dose observations. Body weight measurements, physical examinations, clinical pathology, urinalysis, faecal occult blood (FOB) and electrocardiographic (ECG) and blood pressure measurements, buccal mucosal bleeding time (BMBT), ophthalmology and gastroduodenal endoscopy examinations were conducted throughout the study. At study completion, all dogs were subjected to gross necropsy. Histopathology was performed on selected tissues from all animals in all groups.

**Results:**

No clinical signs were noted, and no toxicologically relevant dose-associated effects were observed.

**Conclusions:**

Results show that Daxocox® is well-tolerated and has a broad safety margin when administered as directed in dogs.

## Background

Nonsteroidal anti-inflammatory drugs (NSAIDs) are widely used for the treatment of inflammatory and pain conditions both in humans and in veterinary patients [4], and are considered a medical cornerstone of osteoarthritis (OA) management. Pain associated with canine OA is often of a chronic nature, and there are evidences that long-term use of NSAIDs in osteoarthritic dogs is more efficacious as compared to short-term intermittent treatments [12].

The different NSAIDs available in veterinary medicine seem to be of similar efficacy, regardless of their cyclooxygenase (COX) selectivity or safety profile [15]. However, most adverse effects (AEs) associated to the use of NSAIDs are related to prostaglandin inhibition, and include gastrointestinal irritation, protein-losing enteropathy, renal damage, and prolonged bleeding time due to prevention of platelet aggregation [15]. In dog clinical trials, the most common AEs described after the use of NSAIDs are vomiting, diarrhoea, anorexia, lethargy and melena, with disturbance of the renal and hepatic systems also being reported [4, 10, 16]. Although it seems that the incidence of AEs in long term treatments of OA with NSAIDs does not increase significantly with time [12], the advent of potential AEs is always a concern for the veterinary practitioner, and should always be monitored [13, 16, 17, 30].

By avoiding or greatly reducing COX-1 inhibition, the aim of COX-2 selective inhibitors is to reduce the incidence of disorders associated to the suppression of the homeostatic prostaglandin synthesis [13, 22]. Still, NSAID induced AEs are related not only to the safety profile of the drug itself, but also to the administered dose, the dose interval, and the route of administration [14, 15]. This stresses the need for overdose safety studies to assure an adequate margin of safety [27].

**Fig. 1:**
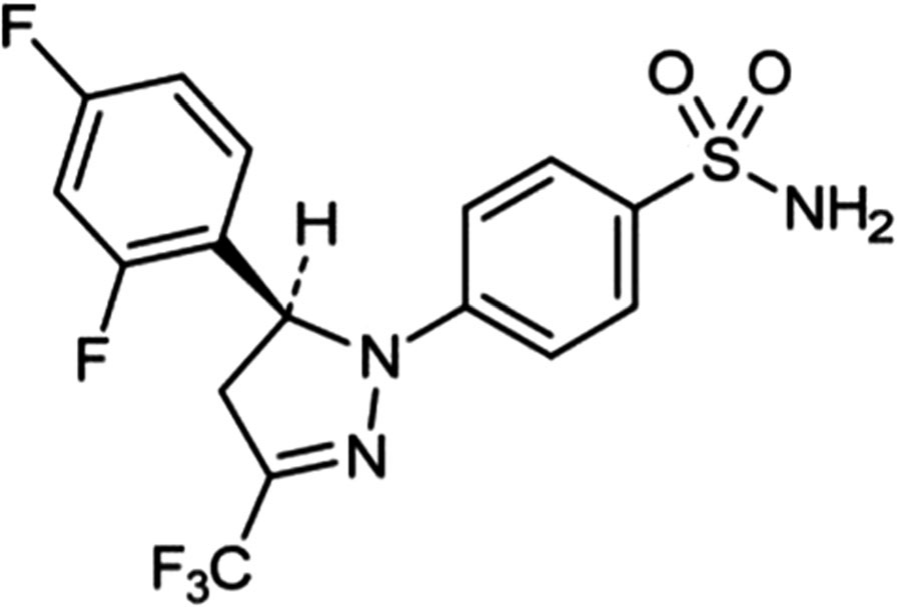
Chemical structure of enflicoxib

Enflicoxib (also known by its research acronym E-6087) is a new pyrazoline derivative COX-2 inhibitor that has been developed for veterinary use in dogs (Fig. 1). It shows potent anti-inflammatory and analgesic activity when tested in experimental models of inflammation and pain [29]. After oral absorption, the pyrazoline ring of enflicoxib is oxidized forming an also active pyrazol metabolite [28]. Pharmacokinetic studies suggest that the long-term activity of enflicoxib would rely mainly on its pyrazol metabolite, which shows a longer elimination half-life and a greater volume of distribution compared to enflicoxib [19, 28]. Enflicoxib has shown to be more than 100-fold selective for COX-2 versus COX-1. In the case of the pyrazol metabolite, the selectivity for COX-2 over COX-1 is > 250-fold, as determined in COX-2 enzymes isolated from sheep placenta and in COX-1 enzyme isolated from rat seminal vesicles [11, 29].

The commercial use of enflicoxib has recently been approved in Europe as Daxocox® tablets for dogs (Ecuphar NV/Animalcare) for the treatment of pain and inflammation associated with OA in dogs, administered at a weekly dose of 4 mg/kg with an initial loading dose of 8 mg/kg [28]. According to current veterinary legislation, a Target Animal Safety (TAS) study conducted with the final commercial product is required to obtain a marketing authorization. Therefore, the purpose of the present study was to investigate the long-term safety of enflicoxib in dogs when administered orally at weekly doses, at the therapeutic dose (4 mg/kg; 1X), and when overdosed at three (3X) and five-times (5X) the therapeutic dose (12 and 20 mg/kg, respectively). To confirm the adequate margin of safety of the product when used in long-term treatments, a dosing period of 7 consecutive months (32 weeks) was chosen for the 1X and 3X treatment groups. The treatment period of the 5X overdose group was established at 3 months (13 weeks) to determine the possible onset of subclinical alterations, if any, only detectable at necropsy, and thereby would provide more accurate information about any possible accidental overdose.

## Materials and methods

### Standards

This study was conducted in accordance with the requirements of current internationally recognized Good Laboratory Practice (GLP) standards as set forth in the UK GLP [25] and OECD [18] Regulations and Directive 2004/10/EC [5], and was designed to meet the requirements of the TAS guideline for veterinary pharmaceutical products [27]. The study was reviewed and approved by the study site Institutional Animal Care and Use Committee and it followed the UK Animals (Scientific Procedures) Act [24].

The number of animals used was the minimum that is consistent with scientific integrity and regulatory acceptability, consideration having been given to the welfare of individual animals in terms of the number and extent of procedures carried out on each animal. This manuscript was prepared in compliance with the ARRIVE guidelines for reporting animal in vivo experiments.

### Animals and maintenance

A total of 32 (16 males and 16 females) healthy, pure bred naïve Beagle dogs, of 8 to 10 months of age were obtained from a laboratory animal supplier (Marshall BioResources, Huntingdon, UK). On arrival, each animal was uniquely identified by a microchip and acclimatized for 28 days before study initiation. Animals were properly vaccinated and dewormed. Following clinical pathology evaluation and a detailed veterinary examination, all animals were considered suitable to be included in the study.

All dogs were housed in pairs of the same sex and dose group in several adjacent indoor pens with softwood-based granules and paper shavings under temperature and humidity-controlled conditions (15 to 24 °C, 40–70% RH). Artificial lighting was provided for approximately 12 h per day. The pens were designed in accordance with the requirements of the UK Home Office Code of Practice for the Housing and Care of Animals [26], and identified with the study code, the treatment group, and the occupants. For each group, periods of exercise and socialisation were permitted daily. Animals were fed individually with 400 g daily of a certified commercial pelleted diet. On dosing days, food was offered immediately after dosing; on non-dosing days, food was offered at approximately the same time as the previous dosing day. Each dog was allowed a minimum of 1 h feeding time. Any uneaten food was removed and subsequently weighed and discarded. Water was available ad libitum.

### Allocation to groups

This was a randomized, controlled study with a parallel design. Before pre-treatment observations, dogs were stratified by sex and weight (males weighing 6.7 to 9.9 kg and females weighing 5.3 to 7.0 kg), and allocated to groups using a pseudo-random body weight stratification procedure, which yielded four groups of eight dogs (four of each sex) with approximately equal mean body weight. The placement of litter mates within the same group was avoided, where possible.

Each study group was randomly allocated to an experimental treatment: Group 1X received the recommended therapeutic dose for the treatment of pain and inflammation associated with osteoarthritis in dogs (8 mg/kg on the first day, as loading dose, and 4 mg/kg weekly thereafter, as maintenance dose); Group 3X received three-times the recommended therapeutic dose (24 mg/kg as loading dose and 12 mg/kg as maintenance dose); Group 5X received five-times the recommended therapeutic dose (40 mg/kg as loading dose and 20 mg/kg as maintenance dose); Group 0X received placebo tablets with similar appearance as the investigational drug at the same weekly intervals, and acted as negative control group.

The allocation of study groups to experimental treatments was blinded to personnel conducting observations to the animals, processing/analysing blood, urine or faecal samples or performing necropsy examinations (it was only known to the Study Director and the Study personnel preparing/administering the doses).

### Investigational veterinary product and administration

The investigational veterinary product was supplied as its final commercial formulation tablets containing 15, 30, 45, 70 or 100 mg of enflicoxib (Daxocox^®^ tablets for dogs, Ecuphar NV/Animalcare). The number and size of the tablets to be administered to each dog on each dosing occasion was calculated based on the most recent body weight and the target dose level. The control animals received three placebo tablets per animal and dosing occasion, independently of body weight. Tablets were administered once a week, immediately before feeding to maximise absorption, according to available PK data [28]. Dogs were administered for a total of 13 weeks in the 5X group, and for 32 weeks in the 0X, 1X and 3X groups.

### Measurements and variables recorded

During the treatment period, clinical condition, body weight, food consumption, ophthalmoscopy, electrocardiography (ECG), blood pressure, haematology (peripheral blood), blood chemistry, urinalysis, faecal occult blood (FOB), endoscopy, buccal mucosal bleeding time (BMBT), organ weights, and macroscopic as well as microscopic investigations were undertaken.

Throughout the study, all animals were observed at least twice daily for general health, mortality, or reaction to treatment, and pens were also inspected for the presence of vomit, the appearance of faeces (consistency/colour and mucus/blood), abnormal urination (none/excessive), and to ensure there were no changes in demeanour/behaviour of the animals overnight. On dosing days, detailed observations were conducted pre-dose, immediately post-dose, 1–2 h after dosing and as late as possible in the working day. Physical examinations were conducted weekly throughout the study on each animal to assess their general overall health. These examinations included, but were not limited to, general condition and behaviour in relation to the main body systems (ocular, musculoskeletal, cardiovascular, reproductive, lymphatic, nervous, integumentary, respiratory, urinary, gastrointestinal). Body weight was measured weekly throughout the study and before necropsy. Food consumption was recorded daily.

Ophthalmologic examinations were performed pre-treatment and at week 13 on all animals, and at week 30 on animals of the 0X, 1X and 3X groups, by means of a binocular indirect ophthalmoscope. Prior to each examination, the pupils of each animal were dilated using tropicamide ophthalmic solution (Mydriacyl 1%^®^, Alcon). The adnexa, conjunctiva, cornea, sclera, anterior chamber, iris (pupil dilated), lens, vitreous and fundus were examined.

Electrocardiograph tracings were recorded for the three standard limb leads (I, II and III) and the three augmented limb leads (aVR, aVL and aVF). The traces were examined visually for any abnormalities of the electrical complexes and the heart rate was recorded. Wave intervals PR, QRS, QT, QTc were calculated. These observations were conducted at pre-treatment (one occasion), and at 3 h and 4 days after dosing on weeks 4 and 13 on all dogs and at week 30 on dogs of the 0X, 1X and 3X groups. For these recordings, animals were placed in a body harness in a restraining frame designed for the purpose. The height of the harness was adjusted so that the animal’s feet were touching the table. The ECG electrodes were attached to the skin by metal clips to a shaved part of the lateral proximal area of each limb. Once the dogs were quiet, recordings started, with a minimum of 2 min of continuous electronic trace recorded. At the same time points, indirect blood pressure tracings were obtained from the infrared photo plethysmograph built into a cuff secured around the base of the tail. Values were obtained for systolic, diastolic and mean arterial pressures using Beatscope Easy software. Pulse rate was also recorded.

Endoscopy was performed pre-treatment on all animals and at week 13 on dogs of the 0X, 1X and 3X groups. Animals of the 5X group were necropsied at this time point. Following anaesthesia, a flexible endoscope was used to examine the oesophageal and gastric (cardia, fundus, lesser curvature and pyloric antrum) mucosa for any sign of hyperaemia or vascularity, dilation, friability, oedema, discoloration, haemorrhage or erosion/ulcer as described by the WSAVA Gastrointestinal Standardization Group [31].

Buccal mucosal bleeding time (BMBT) was assessed pre-treatment and on weeks 4, 8 and 13 on all dogs and on week 30 on dogs of the 0X, 1X and 3X groups. The upper lip was folded up and a lancet placed onto the buccal mucosa and triggered. A timer was started once the incision started freely bleeding and stopped once it was no longer bleeding. Excess blood was blotted using filter paper, but the incision was not touched to avoid disrupting the clot. The time until bleeding was no longer observed was recorded up to a maximum of 15 min.

Blood samples were collected from the jugular vein and a complete panel of clinical pathology endpoints determined according to VICH GL43 at pre-treatment and prior to dosing on weeks 4, 8 and 13 on all animals and on weeks 17, 21, 25 and 30 on animals of the 0X, 1X and 3X groups. Analysis of collected blood samples included a complete blood cell count with determination of haematocrit (Hct), haemoglobin concentration (Hb), erythrocyte count (RBC), absolute reticulocyte count (Retic), mean cell haemoglobin (MCH), mean cell haemoglobin concentration (MCHC), mean cell volume (MCV), red cell distribution width (RDW), total leucocyte count (WBC), differential leucocyte count: neutrophils (N), lymphocytes (L), eosinophils (E), basophils (B), monocytes (M), large unstained cells (LUC) and platelet count (Plt); serum biochemical analysis, including alkaline phosphatase (ALP), alanine aminotransferase (ALT), aspartate aminotransferase (AST), gamma-glutamyltransferase (gGT), lactate dehydrogenase (LDH), creatine kinase (CK), amylase (AMYL), total bilirubin (Bili), urea, creatinine (Creat), glucose (Gluc), total cholesterol (Chol), triglycerides (Trig), sodium (Na), potassium (K), chloride (Cl), calcium (Ca), inorganic phosphorus (Phos), magnesium (Mg), total protein (Total Prot), albumin (Alb) and total bile acids (BiAc); and blood coagulation profile including prothrombin time (PT), activated partial thromboplastin time (APTT), and fibrinogen concentration (Fib).

For haematology, 0.5 mL of blood was collected into tubes containing EDTA K_2_. For serum biochemical analysis, 0.7 mL of blood was collected into serum separator tubes containing lithium heparin as anticoagulant. Additionally, 0.5 mL of blood was collected into tubes containing citrate anticoagulant for the coagulation profile.

At the same timepoints, faecal and urine samples were collected overnight using individual metabolism cages. Analysis of collected urine samples included visual examination of urine for colour, clarity, and volume; use of a refractometer for measurement of urine specific gravity; and use of a urine chemistry analyser for measurement of urine pH and detection of protein, ketones, bilirubin, occult blood, urobilinogen, creatinine and glucose. Urine sediment was examined by microscopy for the presence of cells and crystals. Faecal samples were analysed for the presence of blood with a qualitative method based on the peroxidase-like activity of haemoglobin or its iron-containing degradation products (Beckman Coulter Hemoccult® kit).

Blood samples (2 mL) were also collected at several time points throughout the study for the determination of blood concentrations of enflicoxib and its active metabolite. Toxicokinetic analysis results are reported elsewhere.

### Pathological examination

At the end of their corresponding treatment period all animals were euthanised by an intravenous administration of an overdose of sodium pentobarbitone solution (200 mg/mL) followed by exsanguination and were subjected to a detailed necropsy by a veterinary pathologist. According to guideline VICH GL43, organs were removed, examined, weighed (paired organs were weighed together), and a full complement of tissues was collected from all animals for histological examination: skin with mammary glands (caudal area), adrenals, bone marrow smear, brain (cerebellum, cerebrum, midbrain), cecum, colon, duodenum, epididymides, eyes, femur and marrow (femorotibial joint), gall bladder, heart (including auricular and ventricular regions), ileum, jejunum, kidneys, liver (section from two main lobes), lungs (section from two major lobes including bronchi), lymph nodes (mandibular, mesenteric, left axillary), ovaries, pancreas, Peyer’s patches, pituitary, prostate, skeletal muscle, spinal cord (transverse and longitudinal sections at the cervical thoracic and lumbar levels), spleen, stomach, testes, thymus, thyroid with parathyroids, urinary bladder and uterus with cervix. Any abnormality in the appearance or size of any organ (external and cut surface) was recorded and the required tissue sampled. Tissues were routinely preserved in 10% neutral buffered formalin except for testes and eyes preserved in modified Davidson’s fluid and Davidson’s fluid, respectively, and bone marrow smears that were air dried and subsequently fixed in methanol. Paraffin sections were stained with haematoxylin and eosin and analysed for all animals in 0X, 3X and 5X groups, as well as any abnormality found in the 1X group. Organ weights were recorded for all animals and organ weight ratios relative to body weight were calculated. Findings were either reported as “present” or assigned a severity grade (minimal, slight, moderate, marked, or severe) [9]. A pathologist, who was blinded with respect to individual animal dosing group assignment, undertook a peer review of the microscopic findings.

### Statistical analysis of data

All statistical analyses were carried out using the individual animal as the basic experimental unit, at each timepoint and each treated group versus the control group.

For continuous data, Bartlett’s test was first applied to test the homogeneity of variance between the groups at 1% level [3]. Using tests dependent on the outcome of Bartlett’s test, treated group values (Groups 1X, 3X, or 5X) were compared with data from the control group (Group 0X), incorporating adjustment for multiple comparisons where necessary. Williams’ test [32, 33] or when the F_1_ approximate test for monotonicity of dose-response or trend was significant, suggesting that the dose response was not monotone, Dunnett’s test was performed [6, 7]. If Bartlett’s test was still significant at the 1% level following both logarithmic and square-root transformations, a non-parametric analysis (H_1_ approximate test) was performed. If the H_1_ approximate test for monotonicity of dose-response was not significant at the 1% level, Shirley’s test for a monotonic trend was applied [20]. If the H_1_ approximate test was significant, suggesting that the dose-response was not monotone, Steel’s test was performed instead [23].

For clinical pathology data, if 75% of the data (across all groups) were the same value, Fisher’s exact tests were performed [8]. Treatment groups were compared using pairwise comparisons of each treatment group against the control group.

For organ weight data, analysis of covariance was performed using terminal body weight as covariate [1], unless non-parametric methods were applied. The treatment comparisons were made on adjusted group means to allow for differences in body weight which might influence the organ weights.

Statistical analyses were conducted using SAS^®^ (Statistical analysis system, Version 8.2, Cary, North Carolina: SAS Institute Inc.). Significant differences between the groups compared were considered at the 5% (*p* < 0.05) or 1% (*p* < 0.01) level.

## Results

### Investigational veterinary product doses

Dosing was readily achieved and appeared to be well tolerated by all dogs. The actual mean dose levels administered to the groups receiving 1X, 3X and 5X the maintenance recommended dose of enflicoxib were 5.1, 12.0, and 20.1 mg/kg/week, respectively. Individual administered enflicoxib doses reached or exceeded the intended oral dose levels in all treatment groups, and particularly in the therapeutic dose group (up to 30% overdose), as recommended by current guidelines [27].

### Clinical assessments

There was no mortality and no serious adverse events were reported in any animal during the 32 weeks of treatment. There were no treatment-related adverse findings on clinical observations, ECG, blood pressure or ophthalmologic assessments, in any dog. All dogs appeared healthy throughout the study and until termination. Variable episodes of loose faeces were described in the pen observations for most animals of all treatment groups, including the control group. The incidence and chronology of these episodes showed no relation to treatment and were also observed in the pre-treatment period. Two dogs of the control group and two of the 5X group had sporadic and short episodes of watery faeces. Emesis was also observed on a single occasion in two dogs of the control group and in two dogs of the 5X group. No blood was seen in any dog stools.

Final body weight was similar in all groups, ranging from 8,2 to 11,5 kg in males and from 6,6 to 9,2 kg in females, with an average 18% weight gain during the study. Food consumption was similar between control and treated groups throughout the study. No statistically significant treatment-related differences in either male or female dogs was observed in these parameters (Table 1).

**Table 1:** Summary of mean values of food consumption and body weight changes for healthy Beagle dogs treated with enflicoxib orally once a week for up to 7 months (SD)

Treatment group		Weeks	0X	1X	3X	5X	
Dose (loading+maintenance)			(Placebo)	(8 + 4 mg/kg)	(24 + 12 mg/kg)	(40 + 20 mg/kg)	P^a^
Food consumption (g/animal/day)	Males	1 to 13	298 (53)	297 (47)	271 (48)	306 (36)	ns
		1 to 32	305 (51)	309 (51)	273 (46)	–	ns
	Females	1 to 13	243 (52)	222 (26)	226 (34)	244 (44)	ns
		1 to 32	251 (52)	240 (18)	233 (30)	–	ns
Body weight increase (Kg)	Males	1 to 13	1,3 (0,3)	1,1 (0,5)	1,1 (0,2)	1,5 (0,3)	ns
		1 to 32	1,8 (0,2)	1,1 (0,2)	1,7 (0,1)	–	ns
	Females	1 to 13	0,5 (0,3)	0,2 (0,5)	0,6 (0,4)	0,7 (0,7)	ns
		1 to 32	1,4 (0,9)	0,9 (0,6)	1,0 (0,4)	–	ns

^a^Treated groups compared with 0X group using Williams’ test (ns: *p* > 0.05, * *p* < 0.05, ** *p* < 0.01)

*N* = 4 animals/sex/treatment group

The ophthalmologic examinations revealed no treatment related alterations in any dog. The ECG revealed increased heart rate at several time points during treatment, with values ranging from 104 to 116 bpm, but they were of similar magnitude across all treatment groups and lacked statistical significance. Likewise, disperse statistically significant increases on PR, QRS and QTcF interval or QT interval decreases were noted. However, when compared with the control group values there was no indication of a treatment-related effect on these ECG-intervals nor in the waveform morphology.

Blood pressure and cardiovascular recordings were normal in all dogs, and no differences compared to the control group values were detected. All treatment groups showed similar mean systolic, diastolic, and mean arterial blood pressure values, as well as heart and pulse rate values.

The week 13 endoscopy investigations revealed mild hyperaemia of distal oesophagus and mild erosion/ulcer or haemorrhage (with very small spots of blood specks) in the cardia, fundus and body of the stomach, with diffuse circular red patches on stomach lining, in 3 animals in the 1X group and 2 animals in the 3X group. Similar findings were also observed in two animals of the control group as well as in several animals at pre-treatment examination. All these observations were of a very minor nature and considered to be iatrogenic or background.

No treatment-related effects were observed in BMBT, in either sex, at any dose level. Mean BMBT values were similar between the control and the treated groups.

### Clinical pathology

Summary data for selected haematology, biochemistry and coagulation parameters are presented in Tables 2, 3 and 4. Data are presented combined for males and females as no sex differences were observed.

**Table 2:** Selected haematology parameters mean values for healthy Beagle dogs treated with enflicoxib orally once a week for 7 months (SD)

Treatment group		0X	1X	3X	5X	
Dose (loading+maintenance)		(Placebo)	(8 + 4 mg/kg)	(24 + 12 mg/kg)	(40 + 20 mg/kg)	P^1^
Red blood cell count	Basal	6,5 (0,6)	6,5 (0,4)	6,9 (0,5)	6,8 (0,3)	ns
(× 10^12^/L) (RBC)	Week 4	6,2 (0,6)	6,3 (0,4)	6,2 (0,3)	6,2 (0,3)	ns
	Week 8	6,6 (0,3)	6,4 (0,5)	6,3 (0,3)	6,2 (0,2)	ns
	Week 13	6,6 (0,4)	6,1 (0,4)	6,3 (0,5)	6,1 (0,4)	ns
	Week 17	6,8 (0,5)	6,4 (0,4)	6,7 (0,7)		ns
	Week 21	6,6 (0,7)	6,8 (0,4)	6,5 (0,4)		ns
	Week 25	6,4 (0,2)	6,3 (0,5)	6,4 (0,3)		ns
	Week 30	6,8 (0,4)	6,6 (0,5)	6,7 (0,5)		ns
Reticulocytes	Basal	0,019 (0,005)	0,023 (0,008)	0,020 (0,008)	0,029 (0,011)	ns
(×10^12^/L) (Retic)	Week 4	0,032 (0,010)	0,029 (0,013)	0,003 (0,008)	0,004 (0,015)	ns
	Week 8	0,047 (0,022)	0,003 (0,015)	0,020 (0,007)	0,034 (0,015)	ns
	Week 13	0,022 (0,005)	0,021 (0,007)	0,016 (0,006)	0,026 (0,015)	ns
	Week 17	0,037 (0,012)	0,035 (0,012)	0,029 (0,006)		ns
	Week 21	0,049 (0,014)	0,046 (0,022)	0,038 (0,006)		ns
	Week 25	0,028 (0,021)	0,036 (0,020)	0,027 (0,005)		ns
	Week 30	0,033 (0,009)	0,038 (0,016)	0,022 (0,022)		ns
Hemoglobin (g/dL)	Basal	14,7 (1,1)	14,7 (1,0)	15,5 (1,1)	14,9 (0,8)	ns
(Hb)	Week 4	14,2 (1,2)	14,3 (0,8)	14,1 (1,0)	13,9 (0,7)	ns
	Week 8	14,8 (0,8)	14,5 (0,9)	13,9 (0,8)	13,7 (0,3)	ns
	Week 13	15,3 (1,0)	14,2 (1,0)	14,6 (1,1)	14,1 (1,0)	ns
	Week 17	15,7 (1,1)	14,9 (0,8)	15,3 (1,6)		ns
	Week 21	15,4 (1,4)	15,8 (0,6)	15,3 (1,1)		ns
	Week 25	14,8 (0,6)	14,6 (1,3)	14,8 (0,8)		ns
	Week 30	15,6 (1,0)	15,2 (1,2)	15,3 (1,2)		ns
Hematocrit (L-L)	Basal	0,49 (0,04)	0,49 (0,03)	0,51 (0,03)	0,50 (0,02)	ns
(Hct)	Week 4	0,45 (0,04)	0,45 (0,03)	0,44 (0,03)	0,44 (0,02)	ns
	Week 8	0,48 (0,02)	0,46 (0,03)	0,45 (0,03)	0,44 (0,01)	ns
	Week 13	0,48 (0,03)	0,44 (0,03)	0,45 (0,03)	0,44 (0,03)	ns
	Week 17	0,49 (0,04)	0,46 (0,03)	0,47 (0,06)		ns
	Week 21	0,46 (0,05)	0,48 (0,03)	0,46 (0,03)		ns
	Week 25	0,45 (0,02)	0,44 (0,03)	0,45 (0,02)		ns
	Week 30	0,47 (0,03)	0,46 (0,04)	0,46 (0,04)		ns

^a^Treated groups compared with 0X group using Williams’ test, (ns: p > 0.05, * p < 0.05, ** < 0.01)

N = 4 animals/sex/treatment group

**Table 3:** Selected blood chemistry parameters mean values for healthy Beagle dogs treated with enflicoxib orally once a week for up to 7 months (SD)

Treatment group		0X	1X	3X	5X	
Dose (loading+maintenance)		(Placebo)	(8 + 4 mg/kg)	(24 + 12 mg/kg)	(40 + 20 mg/kg)	P^a^
Alkaline phosphatase (U/L)	Basal	73 (24)	65 (11)	63 (9)	60 (15)	ns
(ALP)	Week 4	103 (46)	70 (16)	86 (37)	88 (27)	ns
	Week 8	95 (35)	74 (19)	87 (32)	125 (39)	ns
	Week 13	99 (34)	74 (23)	88 (34)	95 (18)	ns
	Week 17	97 (34)	75 (25)	86 (31)		ns
	Week 21	95 (41)	77 (26)	87 (40)		ns
	Week 25	88 (34)	69 (28)	85 (33)		ns
	Week 30	86 (41)	76 (31)	84 (37)		ns
Alanine aminotransferase (U/L)	Basal	24 (7)	24 (6)	32 (17)	30 (9)	ns
(ALT)	Week 4	27 (6)	30 (6)	30 (5)	33 (8)	ns
	Week 8	30 (6)	30 (6)	30 (6)	31 (7)	ns
	Week 13	35 (11)	35 (8)	37 (4)	39 (10)	ns
	Week 17	35 (11)	33 (6)	35 (7)		ns
	Week 21	30 (9)	31 (5)	34 (7)		ns
	Week 25	30 (8)	32 (8)	35 (7)		ns
	Week 30	35 (10)	33 (6)	35 (7)		ns
Aspartate aminotransferase (U/L)	Basal	34 (7)	30 (6)	32 (5)	34 (6)	ns
(AST)	Week 4	36 (9)	34 (4)	37 (6)	39 (11)	ns
	Week 8	34 (7)	32 (3)	34 (6)	34 (6)	ns
	Week 13	40 (8)	35 (5)	48 (5)	40 (6)	ns
	Week 17	41 (10)	37 (5)	39 (9)		ns
	Week 21	35 (6)	36 (4)	38 (6)		ns
	Week 25	35 (5)	35 (6)	42 (7)		ns
	Week 30	37 (8)	39 (5)	42 (5)		ns
Urea (mmol/L)	Basal	5,5 (0,7)	5,5 (0,6)	5,8 (1,1)	5,5 (0,8)	ns
	Week 4	5,8 (0,9)	6,4 (0,9)	7,4 (1,0)	7,9 (1,7)^*^	
	Week 8	5,8 (1,1)	6,5 (1,3)	7,3 (1,0)	8,3 (1,8)^*^	
	Week 13	4,6 (0,7)	5,0 (0,5)	6,0 (0,9)^*^	6,9 (0,9)^**^	
	Week 17	6,4 (0,8)	7,3 (1,3)	8,4 (2,1)^*^		
	Week 21	6,3 (1,2)	6,1 (0,9)	7,4 (0,9)		ns
	Week 25	5,7 (0,6)	6,3 (1,0)	7,2 (1,3)^*^		
	Week 30	5,4 (0,7)	6,5 (0,8)	7,2 (1,2)^**^		
Creatinine (μmol/L)	Basal	51 (6)	52 (7)	59 (8)	58 (7)	ns
	Week 4	54 (3)	54 (5)	58 (7)	59 (6)	ns
	Week 8	53 (9)	56 (5)	58 (7)	60 (6)	ns
	Week 13	54 (4)	50 (10)	55 (9)	60 (8)	ns
	Week 17	54 (4)	51 (7)	55 (9)		ns
	Week 21	56 (7)	53 (7)	59 (10)		ns
	Week 25	53 (3)	53 (7)	57 (7)		ns
	Week 30	60 (4)	58 (6)	63 (10)		ns
Total Protein (g/L)	Basal	55 (3)	54 (2)	55 (3)	54 (1)	ns
	Week 4	57 (3)	54 (3)	53 (4)	52 (2)	ns
	Week 8	53 (3)	53 (2)	51 (3)	51 (2)	ns
	Week 13	58 (3)	54 (3)	55 (2)	54 (1)	ns
	Week 17	57 (3)	54 (1)	53 (3)		ns
	Week 21	56 (3)	54 (3)	52 (3)		ns
	Week 25	58 (2)	56 (3)	53 (3)		ns
	Week 30	58 (2)	55 (4)	53 (3)		ns
Albumin (g/L)	Basal	32 (2)	32 (1)	32 (2)	32 (2)	ns
	Week 4	32 (2)	31 (1)	30 (2)	30 (2)	ns
	Week 8	32 (1)	32 (2)	31 (2)	30 (1)	ns
	Week 13	34 (2)	31 (2)	32 (2)	32 (1)	ns
	Week 17	33 (2)	32 (2)	33 (2)		ns
	Week 21	33 (1)	32 (2)	31 (2)		ns
	Week 25	32 (1)	31 (2)	30 (2)		ns
	Week 30	34 (1)	32 (2)	31 (2)		ns
Cholesterol (mmol/L)	Basal	3,6 (0,6)	4,0 (0,5)	3,3 (0,4)	3,5 (0,4)	ns
	Week 4	4,3 (0,5)	4,9 (0,9)	4,4 (0,7)	5,1 (0,8)	ns
	Week 8	4,0 (0,6)	4,8 (0,7)	4,4 (0,7)	5,4 (1,4)	ns
	Week 13	4,1 (0,6)	4,4 (0,7)	5,1 (1,2)^*^	5,5 (1,1)^**^	
	Week 17	3,5 (0,6)	3,9 (0,6)	4,4 (1,0)		ns
	Week 21	4,2 (0,9)	4,8 (0,8)	4,6 (0,7)		ns
	Week 25	4,4 (0,9)	4,7 (0,9)	4,4 (0,7)		ns
	Week 30	3,9 (0,7)	4,7 (1,3)	4,6 (0,7)		ns

^a^Treated groups compared with 0X group using Williams’ test (ns: *p* > 0.05, * *p* < 0.05, ** < 0.01)

N = 4 animals/sex/treatment group

**Table 4:** Coagulation parameters mean values for healthy Beagle dogs treated with enflicoxib orally once a week for up to 7 months (SD)

Treatment group		0X	1X	3X	5X	
Dose (loading+maintenance)		(Placebo)	(8 + 4 mg/kg)	(24 + 12 mg/kg)	(40 + 20 mg/kg)	P^a^
Prothrombin time (sec) (PT)	Basal	8,01 (0,35)	7,85 (0,53)	7,65 (0,39)	7,73 (0,34)	ns
	Week 4	8,13 (0,48)	7,89 (0,58)	7,65 (0,57)	7,68 (0,45)	ns
	Week 8	8,28 (0,35)	8,38 (0,57)	7,96 (0,52)	7,84 (0,40)	ns
	Week 13	8,15 (0,39)	8,05 (0,43)	7,68 (0,39)	7,78 (0,36)	ns
	Week 17	8,03 (0,44)	7,99 (0,60)	7,84 (0,68)		ns
	Week 21	8,26 (0,40)	8,16 (0,46)	7,99 (0,65)		ns
	Week 25	8,38 (0,42)	8,24 (0,41)	7,78 (0,49)		ns
	Week 30	8,04 (0,23)	7,98 (0,41)	7,61 (0,38)		ns
Activated partial thromboplastin time (sec) (APTT)	Basal	16,1 (1,0)	15,7 (1,4)	16,2 (0,9)	16,0 (0,9)	ns
	Week 4	15,6 (0,9)	15,5 (0,7)	15,6 (0,5)	15,3 (0,8)	ns
	Week 8	16,0 (0,8)	15,6 (0,6)	15,7 (0,6)	15,5 (1,0)	ns
	Week 13	15,8 (0,6)	15,3 (0,7)	15,1 (0,2)	14,9 (0,8)	ns
	Week 17	15,3 (1,0)	15,1 (0,9)	15,3 (1,0)		ns
	Week 21	15,4 (0,6)	14,9 (0,8)	15,2 (0,4)		ns
	Week 25	15,6 (0,7)	15,1 (0,7)	15,2 (0,3)		ns
	Week 30	15,0 (0,6)	14,6 (0,4)	14,7 (0,3)		ns
Fibrinogen concentration (g/L) (Fib)	Basal	2,43 (0,76)	2,10 (0,46)	2,26 (0,61)	1,96 (0,27)	ns
	Week 4	2,22 (0,54)	1,95 (0,52)	2,06 (0,43)	1,77 (0,44)	ns
	Week 8	2,09 (0,59)	1,91 (0,51)	1,85 (0,24)	2,56 (0,75)	ns
	Week 13	1,97 (0,41)	1,93 (0,19)	2,02 (0,22)	1,72 (0,25)	ns
	Week 17	2,01 (0,39)	1,90 (0,26)	1,91 (0,26)		ns
	Week 21	2,40 (0,47)	2,19 (0,27)	2,09 (0,31)		ns
	Week 25	2,20 (0,36)	2,03 (0,19)	1,89 (0,35)		ns
	Week 30	1,90 (0,48)	1,93 (0,20)	2,15 (0,63)		ns

^a^Treated groups compared with 0X group using Williams’ test (ns: *p* > 0.05, * *p* < 0.05, ** *p* < 0.01)

N = 4 animals/sex/treatment group

The haematology values were in general comparable between control and treated animals, with minor changes attributed to normal biological variation and no statistical differences at any sampling time (Table 2). When compared to control group values, blood chemistry investigations revealed, a slight increase in urea concentrations in the 3X and 5X dose groups on week 4. This initial increase remained stable throughout the study, and only sporadically the values slightly exceeded the reference range of the laboratory historical control values (8,2 mmol/L). Mean urea plasma levels in the 1X group were always within the reference range values and remained constant and similar to the control group throughout the study. Higher cholesterol values, when compared to the control group, were observed on week 13 only in groups 3X and 5X. These values were therefore considered to be due to normal biological variation and not related to treatment (Table 3). No other relevant effects were seen on any other blood biochemical parameter at any time point.

Likewise, all coagulation parameters (PT, APTT and Fib) were comparable between control and treated groups at all time points (Table 4).

Urinalysis data did not reveal any clinically or statistically significant differences between dogs dosed with enflicoxib and control animals. Indices of renal function, including urinary creatinine and total protein concentrations, and urine specific gravity, were not affected in any group.

There was no apparent effect of treatment on the presence of FOB during the entire treatment period. Sporadic positive results were observed throughout the treatment period across all study groups, including controls, however, all results were negative at the end of treatment (Table 5). The cause of these positive results could not be identified. However, they most likely were false positive results, as FOB results rarely switch from positive back to negative while treatment continues.

**Table 5:** Summary of faecal occult blood data (number of animals with a positive result) for healthy Beagle dogs treated with enflicoxib orally once a week for up to 7 months

Treatment group	0X	1X	3X	5X
Dose (loading+maintenance)	(Placebo)	(8 + 4 mg/kg)	(24 + 12 mg/kg)	(40 + 20 mg/kg)
Basal	0	0	0	0
Week 4	1	1	0	1
Week 8	3	6	5	4
Week 13	0	0	0	0
Week 17	5	4	3	–
Week 21	0	0	0	–
Week 25	1	0	3	–
Week 30	0	0	0	–

N = 4 animals/sex/group

### Post-mortem evaluation

There was no effect on organ weights after 13 weeks of treatment in the 5X group or after 32 weeks of treatment in the 1X and 3X groups. All observed differences were minor or lacked dose-relationship and, consequently, were attributed to normal biological variation (Table 6).

**Table 6:** Organ weights mean values (g) for healthy Beagle dogs treated with enflicoxib orally once a week for up to 7 months (SD)

Treatment group	0X	1X	3X	5X	
Dose (loading+maintenance)	(Placebo)	(8 + 4 mg/kg)	(24 + 12 mg/kg)	(40 + 20 mg/kg)	P^a^
Terminal body weight (kg)	8,40 (1,44)	8,19 (1,03)	8,39 (1,31)	8,31 (1,30)	ns
Adrenal glands	1,14 (0,48)	1,37 (0,31)	1,13 (0,71)	0,91 (0,75)	ns
Brain	70,25 (6,49)	72,14 (5,47)	72,59 (4,69)	72,05 (5,87)	ns
Epididymides (N = 4)	4,25 (0,98)	3,66 (0,17)	3,51 (0,81)	2,81 (0,30)	ns
Heart	79,83 (12,48)	77,74 (11,12)	80,29 (9,58)	74,69 (13,18)	ns
Kidneys	47,26 (12,79)	43,69 (10,24)	38,94 (7,01)	42,00 (8,61)	ns
Liver	295,58 (51,21)	296,02 (40,98)	288,81 (41,54)	303,38 (54,60)	ns
Pituitary glands	0,07 (0,01)	0,06 (0,01)	0,06 (0,01)	0,06 (0,01)	ns
Prostate	7,48 (1,51)	9,85 (4,23)	6,60 (1,63)	6,41 (1,67)	ns
Spleen	118,70 (48,95)	123,11 (36,63)	129,31 (51,31)	81,52 (29,58)	ns
Testes (N = 4)	13,03 (1,15)	11,92 (0,72)	11,79 (0,75)	14,48 (4,12)	ns
Thymus gland	4,95 (1,33)	4,55 (1,28)	5,43 (1,53)	8,70 (2,27)	ns
Thyroid/parathyroid glands	0,53 (0,10)	0,64 (0,10)	0,54 (0,14)	0,60 (0,11)	ns
Ovaries (N = 4)	0,81 (0,47)	0,28 (0,49)	0,52 (0,52)	1,64 (1,31)	ns
Uterus and Cervix (N = 4)	3,61 (0,48)	3,43 (0,77)	5,75 (5,25)	11,46 (5,74)	ns

^a^Treated groups compared with 0X group using Williams’ test (NS: p > 0.05, * p < 0.05, ** p < 0.01)

N = 4 animals/sex/group

The macroscopic pathology examination did not reveal any treatment related alteration. The incidence and distribution of any finding was sporadic, incidental, of no clinical or toxicological significance and unrelated to dose (Table 7).

**Table 7:** Summary data of macroscopic examination for healthy Beagle dogs treated with enflicoxib orally once a week for up to 7 months

Treatment group	0X	1X	3X	5X
Dose (loading+maintenance)	(Placebo)	(8 + 4 mg/kg)	(24 + 12 mg/kg)	(40 + 20 mg/kg)
Adrenals				
Enlarged	0	0	0	1
Colon				
Dark area(s)	0	0	1	0
Epididymides				
Unilaterally Absent	0	0	1	0
Jejunum				
Abnormal colour	1	0	0	0
Dark area(s)	0	0	0	1
Depression(s)	1	1	0	0
Kidneys				
Abnormal Colour	0	0	0	1
Liver				
Enlarged	0	0	0	1
Lungs and Bronchi				
Abnormal colour	0	0	0	1
Firm	0	0	0	1
Incomplete deflation	0	0	0	1
Pale area(s)	0	0	0	1
Lymph Node, Mesenteric				
Dark	0	0	0	1
Pituitary				
Cyst(s)	0	0	0	1
Spleen				
Abnormal Shape	0	0	0	1
Adhesions	0	0	0	1
Capsule Thickened	0	0	0	1
Stomach				
Dark area(s)	0	0	0	1
Thyroids				
Small	0	0	0	1
Uterus				
Serosal cyst(s)	0	0	0	1

N = 4 animals/sex/group

The histopathological examination performed at week 13 in the 5X group and at week 32 in the other groups did not reveal any treatment related changes. The observed histopathological findings were of undetermined pathogenesis and not dose-related. In particular, the histopathological evaluation of the gastrointestinal tract did not reveal any lesions attributable to treatment.

## Discussion

The gastrointestinal tract is the primary site of NSAID organ toxicity in humans and companion animals [15]. In this study, the lack of significant findings at the endoscopic examination combined with the absence of lesions at necropsy (macro and microscopically) or the lack of faecal occult blood, allow to conclude that enflicoxib oral treatment did not produce any relevant toxicological effects at gastrointestinal level, even when overdosed for an extended period of time. Some episodes of loose (soft-formed) faeces and emesis in some dogs, including control group animals, were recorded. These observations were also present during the pre-treatment period and can be attributable to general stress conditions associated with kennelling, daily handling, and periodically conducted investigation. The absence of changes on bodyweight or feed consumption at any of the tested doses, would further support the lack of treatment related adverse effects at the gastrointestinal level. These results contrast with the well-known adverse effects of most NSAIDs on the gastric mucosa. It has long been acknowledged that some NSAIDs are associated to a higher risk of gastrointestinal toxicosis compared to others and this is partly explained due to their different selectivity profile towards the COX-2 isoenzyme. However, NSAIDs can induce gastric damage through both local and systemic effects, with the local effects being associated to the physical properties of NSAIDs. As most NSAIDs are of a slightly acidic nature, they concentrate within the gastric mucosa through a process known as ion trapping. This can lead to a direct cellular injury that is independent of their systemic effects, with the latter being dependent on the inhibition of prostaglandin synthesis [2, 14]. As enflicoxib is administered only once on a weekly basis, its local concentrations in the gastric mucosa during a treatment course would be much lower and transient compared to other NSAIDs needing a continuous daily oral administration. Therefore, in addition to its COX-2 selectivity, the once weekly posology of the orally administered enflicoxib and the reduced time for local gastrointestinal effects, as compared to other daily administered NSAIDs, could also account for the good gastrointestinal tolerability seen in this study.

PGE_2_ and PGI_2_ function as vasodilatory agents regulating renal blood flow. During an episode of decreased renal perfusion, PGE_2_ and PGI_2_ cause afferent arteriolar dilation, which in turn helps to maintain renal blood flow, counteracting the effect of systemic vasoconstrictors such as vasopressin, angiotensin, and norepinephrine. Clinically, NSAIDs associated renal adverse effects are the primary consequence of decreased PG production. As such, NSAID-induced nephropathy is characterized by papillary necrosis and interstitial nephritis, a condition that has been associated with the use of several different types of NSAIDs [14]. In dogs administered NSAIDs, renal problems may cause reduced glomerular filtration rate which would produce increased blood urea values. Also, episodes of gastrointestinal bleeding would lead to protein loss into the intestinal lumen, with subsequent digestion and enhanced urea production in the liver. In our study, no decrease of total protein or albumin plasma concentrations was detected, and the increased urea values detected in the high dose groups remained stable and largely within the laboratory reference range up to the end of the study. As there were no changes in other blood or urinary parameters suggestive of an effect on the kidneys, nor organ weight changes, macroscopic or microscopic kidney findings at the post-mortem examination, it can be concluded that enflicoxib is well tolerated at renal level.

The blood chemistry evaluations did not reveal relevant changes in plasma liver enzymes concentrations (ALT, AST, ALP) and no macroscopic or histologic abnormalities in the post-mortem analysis were detected. Further to this, enflicoxib administration showed no signs of liver toxicity.

Enflicoxib did not show potential for interference with blood haemostasis, as no changes were observed in the coagulation parameters (PT, APP or Fib), nor in the BMBT.

According to Monteiro-Steagall et al., [16] in dogs treated with NSAIDs, adverse events appeared to be more commonly recorded in clinical trials when compared with research studies, which may be related to the fact that research studies use young healthy animals, in contrast to clinical studies where older dogs with naturally occurring disease are enrolled. This could be considered a limitation of the present study. However, the need for safety extrapolations from the laboratory setting to the target population in the field is overcome by incorporating overdose dosing schemes in TAS studies [21]. Indeed, the international guideline that was followed for this study recommends the administration of an overdose of up to five times the therapeutic dose for an extended period of time [27], which should allow a reasonable extrapolation to the target population.

On the other hand, the duration of the treatment period in our study and the extensive list of parameters studied, which included endoscopy and histology, strengthens the relevance of the study results. The fact that most of the pathology examinations and laboratory determinations were performed blindly to treatment also adds further robustness to the study.

## Conclusions

This study supports the safety of weekly long-term use of Daxocox® tablets at the recommended labelled dose with a margin of safety up to five times the therapeutic dose.

## Data Availability

The data that support the findings of this study are not shared due to confidentiality but are available from the corresponding author upon reasonable request.
